# Angiotensin Receptor Blockers and Cognition: a Scoping Review

**DOI:** 10.1007/s11906-023-01266-0

**Published:** 2023-09-21

**Authors:** Zhen Zhou, Suzanne G. Orchard, Mark R. Nelson, Michelle A. Fravel, Michael E. Ernst

**Affiliations:** 1 School of Public Health and Preventive Medicine, Monash University, 553 St Kilda Road, Melbourne, VIC 3004 Australia; 2 Menzies Institute for Medical Research, University of Tasmania, Hobart, TAS Australia; 3 Department of Pharmacy Practice and Science, College of Pharmacy, The University of Iowa, Iowa, IA USA; 4 Department of Family Medicine, Carver College of Medicine, 01291‐A PFP, The University of Iowa, 200 Hawkins Dr, Iowa, IA 52242 USA

**Keywords:** Anti-hypertensives, Dementia, Alzheimer’s disease, Cognition, Angiotensin II receptor blockers

## Abstract

**Purpose of Review:**

To provide an overview of the association between angiotensin II receptor blocker (ARB) use and cognitive outcomes.

**Recent Findings:**

ARBs have previously shown greater neuroprotection compared to other anti-hypertensive classes. The benefits are primarily attributed to the ARB’s effect on modulating the renin-angiotensin system via inhibiting the Ang II/AT1R pathway and activating the Ang II/AT2R, Ang IV/AT4R, and Ang-(1–7)/MasR pathways. These interactions are associated with pleiotropic neurocognitive benefits, including reduced β-amyloid accumulation and abnormal hyperphosphorylation of tau, ameliorated brain hypo-fusion, reduced neuroinflammation and synaptic dysfunction, better neurotoxin clearing, and blood–brain barrier function restoration. While ACEis also inhibit AT1R, they simultaneously lower Ang II and block the Ang II/AT2R and Ang IV/AT4R pathways that counterbalance the potential benefits.

**Summary:**

ARBs may be considered an adjunctive approach for neuroprotection. This preliminary evidence, coupled with their underlying mechanistic pathways, emphasizes the need for future long-term randomized trials to yield more definitive results.

## Introduction

Dementia presents a significant and growing public health challenge, imposing substantial burdens on individuals, families, and society. It primarily affects older adults, with the risk of dementia doubling every 10 years after age 65 [1]. There are over 55 million people living with dementia worldwide, and approximately 10 million new cases are recorded every year [2]. Unfortunately, there is no known cure for dementia due to its complex and largely irreversible nature. Consequently, efforts geared toward preventing or delaying the progress of dementia in its early stages remain a primary strategy for reducing the burden of the disease. Identifying currently available medications that are associated with lower risks of dementia and cognitive decline is an important ancillary strategy to address this public health issue.

Up to one-third of dementia cases could be attributable to modifiable risk factors [3]. Hypertension, for example, has been demonstrated to accelerate cognitive decline and increase the risk of both vascular dementia and Alzheimer’s disease (AD). The brain, lacking energy reserves, relies on cerebral blood flow to supply necessary nutrients and oxygen. Adequate vascular perfusion is therefore fundamental for maintaining brain energy, homeostasis, and normal functioning. Hypertension can impair neurocognitive function and disrupt normal brain homeostasis by promoting intracerebral inflammation and oxidative stress, reducing cerebral blood flow, and leading to cerebral hypoperfusion via thickening and hardening of the brain arteriole walls, narrowing the lumen, and causing injuries to small vessels [4–7]. Hypertension has also been demonstrated to accelerate the deposition of microvascular β-amyloid and neurofibrillary tangles by promoting inward brain vascular remodelling, increasing blood–brain barrier permeability, enhancing the processing of the β-amyloid protein precursor, and aggravating brain hypoperfusion and neuroinflammation [8]. A recent clinical trial (SPRINT-MIND) found that intensive blood pressure (BP) control improved neurocognitive outcomes, though the results were not statistically significant due to the early termination of the trial [9•].

Numerous studies have demonstrated the benefits of anti-hypertensive medications in reducing the risk of vascular dementia and AD, both in individuals with and without initial cognitive impairment [10, 11]. This benefit is partly attributed to the reversal of the detrimental effects of reduced cerebral blood flow caused by hypertension. Considering the increased risk of dementia with aging and the fact that up to half of individuals of age over 70 have hypertension [12], identifying specific anti-hypertensive medication classes that have the potential to protect against dementia and cognitive decline, while effectively controlling BP and cardiovascular disease (CVD) risk, has become a clinical priority.

Epidemiologic studies have yielded mixed findings on the relative neurocognitive effects of different anti-hypertensive medication classes, with some reporting no significant difference and others showing the opposite [13–15]. Despite the inconsistency, there has been accumulating evidence supporting a greater neuroprotective association with angiotensin II receptor blockers (ARBs) than other classes [16]. ARBs have been proposed in many studies to exert pleiotropic neuroprotective effects beyond BP regulation, including anti-inflammation, anti-oxidation, and anti-proptosis, maintaining the normal vascular and cellular structure and functions of the brain, preventing the accumulation of β-amyloid and reducing the hyperphosphorylation of tau.

To inform clinical practice, this review is intended to summarize the updated evidence from preclinical studies, clinical randomized trials, and observational studies comparing ARB use, with other classes or with no anti-hypertensive medication use, on cognitive outcomes. A particular focus was placed on comparing ARBs with angiotensin-converting enzyme inhibitors (ACEis), considering their similar mechanisms of action and clinical equipoise in BP and CVD risk reduction. The potential pharmacological and pathophysiological mechanisms underlying ARB’s putative neurocognitive benefits are thoroughly discussed. We also reviewed potential risk modifiers of the association between ARBs and neurocognitive outcomes as identified in previous studies. Finally, we highlight evidence gaps and suggest opportunities for future research examining the association between ARBs, dementia, and cognitive decline.

## Postulated Neuroprotective Effects of Renin-Angiotensin System (RAS) Inhibiting Medications

Blockage of the renin-angiotensin system (RAS) lowers BP and is recognized as a useful strategy for preventing incident and recurrent CVD events, including strokes and their sequelae such as vascular dementia (VaD) [17]. ARBs and ACEis are typical RAS-inhibiting anti-hypertensive medications that are widely used in clinical practice. ARBs work by inhibiting the function of type-1 receptors of angiotensin II (AT1Rs), thus diminishing the vessel-constricting effect from the Ang II binding to AT1. ACEis act upstream by blocking the action of angiotensin-converting enzyme 1 (ACE-1), leading to a reduction in downstream Ang II synthesis.

Both ACEi and ARBs have been shown to confer direct and indirect benefits in preventing and delaying cognitive decline and dementia beyond BP regulation [18–22]. ACEi and ARBs have been suggested to enhance neurovascular coupling and induce synaptic plasticity by increasing the release of glutamate to induce the production of growth factors, cytokines, and other intercellular messengers in neurons and glial cells, further enhancing learning and memory processes [23]. Preclinical studies suggested that ACEis may reduce β-amyloid degradation which is catalyzed by the ACE-1 N-terminus, while ARBs catabolize β-amyloid peptides by increasing the levels of proteins involved in its metabolism, including insulin-degrading enzyme, neprilysin, and transthyretin [24]. A handful of studies found a reduction in β-amyloid deposition only in ARB users but not in ACEi users [25–27, 28•]. For example, a recent longitudinal study in cognitively normal older adults (*n* = 142) found that ARB users had slower β-amyloid accumulation in the cortex, specifically in the caudal anterior cingulate and precuneus, and the precentral and postcentral gyri, compared with ACEi users [28•]. However, the strength of the evidence was limited by the small study sample size. A study of 83 individuals with mild cognitive impairment found a lower risk of progression to AD in RAS inhibitor users compared to non-RAS inhibitor users, and brain imaging showed that RAS inhibitor users had fewer neurofibrillary tangles, comprised of hyperphosphorylated microtubule-associated tau protein in multiple brain regions [29].

Although the efficacy of ARBs and ACEis is comparable in BP-lowering and reduction of vascular events, studies have consistently reported better neurocognitive outcomes in ARB users compared with ACEi users [30, 31]. In a large UK case–control study of 48,363 individuals (mean age 82 years) with good adherence to anti-hypertensive therapy, the use of either ARBs or ACEis was associated with a reduced risk of AD compared with the use of other classes, and a stronger inverse association was observed with ARBs than ACEis (odds ratios, 0.47 and 0.76, respectively) [32]. A recent meta-analysis [33••] including 3 million individuals, investigating the association between RAS inhibitor anti-hypertensive medications and dementia risk, found that ARB use was associated with a 22% reduced risk of all-cause dementia and a 26% decreased risk of AD when compared to other classes; while there was no significant difference between ACEIs and other classes, the risk of dementia was 14% lower in ARB users when compared to ACEi. This study, however, did not find any significant benefits from ARBs against vascular dementia, suggesting a greater direct benefit conferred by ARBs on neurodegeneration compared to their systemic vascular benefits. This finding was supported by another case–control study (*n* = 48,363) observing a stronger association of ARB use with AD than with vascular dementia and other dementia types [32].

Not all studies report consistent findings. Some observational studies and early randomized trials suggest no significant neuroprotection with ARB or the use of other anti-hypertensive classes and no difference in the risk of neurological outcomes between ARBs and other classes. For example, two randomized-controlled trials, ONTARGET (the Ongoing Telmisartan Alone and in Combination with Ramipril Global Endpoint Trial) and TRANSCEND (the parallel Telmisartan Randomized Assessment Study in ACE Intolerant Subjects with Cardiovascular Disease), involving 25,620 and 5926 individuals aged 55 years and older with established CVD or diabetes with end-organ damage, respectively, reported neither a significant effect of RAS inhibitor anti-hypertensive agents on cognitive function nor a differential effect between ARBs and ACEis during a median follow-up of 4.7 years [34••]. This neutral effect may be explained by the younger population studied. It must be acknowledged that there remains significant uncertainty regarding the effect of RAS inhibitors on neurological outcomes and the relative effects between different classes, due to the inconsistency of historical evidence. Table 1 shows a summary of key historical studies comparing ARBs with other anti-hypertensive classes or no use of anti-hypertensive medications.

**Table 1:** Key randomized controlled trials and observational studies

Authors	Study type	Study population	Mean (SD) age	Sample size	Follow-up duration	Intervention/comparator	Outcomes	Results
Lithell et al. (SCOPE) [35]	A post hoc analysis of RCT	Elderly patients (70–89 years) with mild-moderate hypertension	76.4 (4.5)	2098	Mean 3.6 years	Intervention: ARB (candesartan) without add-ons Control: placebo without add-ons	Significant cognitive decline; dementia	Significant cognitive decline: 10.5 versus 13.7 cases per 1000 patient-years, *p* > 0.20 Dementia: 6.7 versus 6.0 cases per 1000 patient-years, *p* > 0.20
Skoog et al. (SCOPE) [36]	RCT	Elderly patients (70–89 years) with mild-moderate hypertension and MMSE ≥ 24	76	4937	Mean 3.7 years	Intervention: ARB (candesartan) with/without add-ons Control: placebo with/without add-ons	MMSE score change over time; incident dementia	MMSE score change: patients with low cognitive function (MMSE 24–28): − 0.04 vs. − 0.53, *p* = 0.04 Patients with high cognitive function (MMSE 29–30): − 0.80 vs. − 0.73, NS Dementia: patients with low cognitive function, 4.3% vs. 4.5%, NS Patients with high cognitive function 1.2% vs. 0.7%, NS
Diener et al. (PRoFESS) [37]	RCT	Patients who had an ischaemic stroke	66.1 (8.6)	20,332	Median 2.4 years	Intervention: ARB (telmisartan) Control: placebo	A decrease in MMSE score of 3 points or more; cognitive impairment (MMSE < 24); dementia	A decrease in MMSE score of 3 points or more: RR0.95 (0.87–1.05) Cognitive impairment: 25% vs.25% (*p* = 0.98) Dementia: 5% vs. 5% (*p *= 0.99)
Hanon et al. [38]	Prospective cohort (28 countries)	Hypertensive patients aged ≥ 50 years who were never treated with anti-hypertensive agents	64.2 (9.5)	25,745	Total 0.5 year	Intervention: ARB (eprosartan) with and without add-ons Control: no ARB	MMSE score change between baseline and 6-month follow-up	27.9 vs. 27.1; *p* < 0.0001
Li et al. (US Veteran Affairs) [39••]	Prospective cohort	Adults aged ≥ 65 years with CVD and without dementia; predominantly males	74 (5.5)	786,190	Total 4 years	Intervention: ARB Control: ACEi (lisinopril), other CVD meds)	Incident AD; incident dementia	Incident AD: (1) ARB vs. ACEi: HR 0.81, 95% CI 0.68–0.96, *p* = 0.02; (2) ARB vs. other CVD meds: HR, 0.84, 95% CI, 0.71–1.00, *p* = 0.045 Incident dementia: (1) ARB vs. ACEi: HR 0.81, 95% CI 0.73–0.90, *p* < 0.001; (2) ARB vs. other CVD meds: HR, 0.76, 95% CI, 0.69–0.84, *p* < 0.001
Anderson et al. (ONTARGET) [34••]	RCT	Adults aged ≥ 55 years with CVD or DM with end-organ damage	66.4 (7.2)	25,620	Median 4.7 years	Intervention: ARB (telmisartan) Control: ACEi (ramipril)	Cognitive impairment; cognitive decline; cognitive impairment or decline; cognitive impairment or stroke; cognitive impairment, MMSE < 18	Cognitive impairment: OR, 0.90; 95% CI, 0.80–1.01, *p* = 0.06 Cognitive decline: OR, 0.97; 95% CI, 0.89–1.06, *p* = 0.53 Cognitive impairment or decline: OR, 0.96; 95% CI, 0.89–1.04, *p* = 0.38 Cognitive impairment or stroke: OR, 0.90; 95% CI, 0.81–0.99, *p* = 0.03 Cognitive impairment, MMSE score < 18: OR, 0.84; 95% CI, 0.71–0.99, *p* = 0.04
Anderson et al. (TRANSCEND) [34••]	RCT	Adults aged ≥ 55 years with CVD or DM with end-organ damage who are intolerant to ACEi	66.9	5926	Median 4.7 years	Intervention: ARB (telmisartan) Control: placebo	Cognitive impairment; cognitive decline; cognitive impairment or decline; cognitive impairment or stroke; cognitive impairment, MMSE < 18	Cognitive impairment: OR, 0.97; 95% CI, 0.81–1.17, *p* = 0.76 Cognitive decline: OR, 1.10; 95% CI, 0.95–1.27, *p* = 0.22 Cognitive impairment or decline: OR, 1.08; 95% CI, 0.94–1.24, *p* = 0.27 Cognitive impairment or stroke: OR, 0.96; 95% CI, 0.81–1.13, *p* = 0.60 Cognitive impairment, MMSE score < 18: OR, 1.16; 95% CI, 0.89–1.50, *p* = 0.27
Davies et al. [32]	Case–control study (UK general practice)	Adults aged ≥ 60 years	82.2 (7.0)	9197 (case); 39,166 (control)	-	Intervention: ARB Control: other anti-hypertensive agents	Dementia; probable AD; possible AD; probable VaD; unspecified or other dementia	Any dementia: OR: 0.55, 95% CI, 0.49–0.62, *p* < 0.001 Probable AD: OR: 0.47, 95% CI, 0.37–0.58, *p* < 0.001 Possible AD: OR: 0.51, 95% CI, 0.43–0.61, *p* < 0.001 Probable VaD: OR: 0.70, 95% CI, 0.57–0.85, *p* < 0.001 Unspecified or other dementia: OR: 0.62, 95% CI, 0.47–0.81, *p* < 0.001
Hsu et al. [40]	Prospective cohort	Newly diagnosed hypertensive patients without AD	58 (13)	32,911	Mean 5.2 years	Intervention: ARB Control: no ARB	Incident AD	Adjusted HR: 1.00, 95% CI, 0.88–1.13
Yasar et al. [41]	A post-hoc analysis of RCT (cohort)	Adults ≥ 75 years of age with normal cognition or MCI	78.6 (3.3)	2248	Median 6.1 years	Intervention: ARB Control: other anti-hypertensive agents, no use of anti-hypertensive medications	Incident AD	ARB vs. none: adjusted HR: 0.35, 95% CI, 0.19–0.65, *p* = 0.001 ARB vs. ACEi: adjusted HR: 0.63, 95% CI, 0.32–1.25, *p* = 0.18 ARB vs. diuretic: adjusted HR: 0.75, 95% CI, 0.39–1.42, *p* = 0.37 ARB vs. BB: adjusted HR: 0.55, 95% CI, 0.29–1.05, *p* = 0.07 ARB vs. CCB: adjusted HR: 0.63, 95% CI, 0.33–1.19, *p* = 0.16
Chiu et al. [42]	Prospective cohort (Taiwan National Health Insurance)	Adults aged ≥ 50 years without dementia	62.2 (7.4)	49,062	Total 11 years	Intervention: ARB Control: other anti-hypertensive agents	Dementia; AD; VaD	Dementia: adjusted HR: 0.54, 95% CI (0.51–0.59) AD: adjusted HR: 0.53, 95% CI, 0.43–0.64; VaD: adjusted HR: 0.63, 95% CI, 0.54–0.73
Goh et al. [43]	Prospective cohort (UK primary care practices)	Adults without dementia within the first 12-month follow-up	Median 64.5 (IQR: 54–74)	469,366	Median 4.3 years	Intervention: ever ARB use Control: ACEi only	Incident dementia	Adjusted HR: 0.92, 95% CI, 0.85–1.00, *p* = 0.04
Kuan et al. [44]	Prospective cohort	Adults aged ≥ 50 years with type-2 DM and hypertension	65 (9.6)	3560	Total 12 years	Intervention: ARB Control: no ARB	Incident dementia; AD; VaD	Any dementia: adjusted HR: 0.60, 95% CI, 0.37–0.97 AD: adjusted HR: 0.80, 95% CI, 0.29–2.17 VaD: adjusted HR: 0.41, 95% CI, 0.19–0.89
van Middelaar et al. (preDIVA trial) [45]	Post hoc analysis of RCT	Community-dwelling older adults	74.4 (2.5)	1,951	Median 6.7 years	Intervention: ARB use Control: other anti-hypertensive agents	Incident dementia	Adjusted HR: 0.60 (0.3–-0.98), *p* < 0.05
Barthold et al. [46]	Retrospective cohort (US Medicare insurance)	Adults aged ≥ 65 years	78	1,343,334	NR (2009–2013)	Intervention: ARB use Control: non-RAS anti-hypertensive agents	Incident AD	Male: OR: 0.83 (0.79–0.88) Female: OR: 0.94 (0.91–0.97)
Bohlken et al. [47]	Case–control study	Adults aged ≥ 60 years	80.6 (7.1)	12,405 case; 12,405 control	-	Intervention: ARB Control: no ARB	Dementia	OR: 0.79, 95% CI, 0.73–0.87, *p* < 0.001
Hajjar et al. [48]	RCT	Adults aged ≥ 55 years with MCI and hypertension	66.0 (7.8)	176	Total 1 year	Intervention: ARB (candesartan) Control: ACEi (lisinopril)	Executive function; executive abilities; episodic memory; retention	Executive function: ES = − 12.8; 95% CI, − 22.5 to − 3.1, *p* = 0.01 Executive abilities: ES = − 0.03; 95% CI, − 0.08 to 0.03, *p* = 0.31 Episodic memory: ES = 0.4; 95% CI, 0.02 to 0.8, *p* = 0.04 Retention: ES = 5.1; 95% CI, 0.7 to 9.5, *p* = 0.02
Cohen et al. [49] (SPRINT)	A secondary analysis (cohort study) to clinical trial	Adults not taking an ARB or ACEi at the baseline visit	67 (9.5)	9361	Median 4.9 years	Intervention: ARB Control: ACEi	Amnestic MCI or probable dementia	Overall: HR, 0.93; 95% CI, 0.76–1.13
Lee et al. [50]	Retrospective cohort study using the Korean Health Insurance	Patients diagnosed with ischaemic heart disease	70 (7.0)	57,420	Mean 8.6 years	Intervention: ARB/ACEi Control: no use of ARB/ACEi	Incident AD	ARB vs. no RAS inhibitor users: adjusted HR, 0.94; 95% CI, 0.90–0.99 ACEi vs. no RAS inhibitor users: adjusted HR, 1.03; 95% CI, 0.97–1.10 BBB-crossing ARBs vs. no RAS inhibitor users: adjusted HR, 0.83; 95% CI, 0.78–0.88 BBB-non-crossing ARBs vs. no RAS inhibitor users: adjusted HR, 0.98; 95% CI, 0.93–1.04 BBB-crossing ACEis vs. no RAS inhibitor users: adjusted HR, 1.03; 95% CI, 0.96–1.10 BBB-non-crossing ACEis vs. no RAS inhibitor users: adjusted HR, 1.18; 95% CI, 1.06–1.31

Abbreviations: *ACEi*, angiotensin-converting enzyme inhibitor; *AD*, Alzheimer’s disease; *ARB*, angiotensin-receptor blocker; *BBB*, blood brain barrier; *CI*, confidence interval; *CVD*, cardiovascular disease; *DM*, diabetes mellitus; *ES*, effect size; *HR*, hazard ratio; *MCI*, mild cognitive impairment; *MMSE*, mini-mental state examination; *NR*, not reported; *NS*, not significant; *OR*, odds ratio; *RCT*, randomized controlled trial; *RAS*: renin-angiotensin-system; *VaD*, vascular dementia

## Possible Mechanisms for the Pleiotropic Neuroprotective Benefits of ARBs Independent of Blood-Pressure-Lowering Effect

### Angiotensin Hypothesis

Several plausible explanations have been proposed for the putative superiority of ARBs over ACEis and other classes in neurocognitive outcomes. The “Angiotensin hypothesis” has gained significant attention as a plausible explanation, although much of the evidence comes from animal and laboratory studies [51, 52]. The process begins with renin cleaving the N-terminal of angiotensinogen to produce angiotensin I (Ang I), which is then converted to the bioactive peptide, Ang II, by ACE. Ang II and its derivates in the brain are known to play a role in neurodegeneration by modulating neuroinflammation, oxidative stress, and neuronal apoptosis [52]. Ang II can bind to two receptors, AT1R (Ang II type-1 receptors) and AT2R (Ang II type-2 receptors), which are expressed in multiple sites in the brain, including neurons, astrocytes, oligodendrocytes, and microglia [53]. Activation of AT1R can exert deleterious effects on the brain, including disrupting brain functions and metabolism and damaging cerebral vasculature. Specifically, AT1R activation can lead to vasoconstriction, reduced oxygen and nutrient supply to the brain, activation of neuroinflammation, oxidative stress, and impairment of mitochondrial and cholinergic function. It can also contribute to blood–brain barrier (BBB) dysfunction, brain cell injury, and the retention of β-amyloid, collectively contributing to neurodegeneration [54••, 55].

ACEis and ARBs may confer neuroprotection via antagonizing AT1 receptor activation and blocking the ACE/Ang II/AT1R axis, with ARBs selectively and competitively blocking the AT1R, preventing it from binding to Ang II, and ACEi acting upstream in the ACE pathway. Prevention of the synthesis of AT1R by ARB could further lead to substantial upregulation in Ang II levels as a result of negative feedback regulation. In contrast, ACEis inhibit ACE activity, thus downregulating the production of downstream Ang II. The elevation in circulating Ang II levels with ARBs further promotes the upregulation of the unopposed Ang II/AT2R pathway, which is associated with a wide range of purported neurocognitive benefits. This includes, but is not limited to, the improvement of cerebral hypoperfusion and brain metabolism by increasing the oxygen and nutrient supply to the brain and maintaining brain homeostasis by reducing hypoxia, inflammation, oxidative stress, and cell proptosis. In addition, activation of the Ang II/AT2R pathway may further slow the deposition of β-amyloid, promote axonal regeneration, and improve neurogenesis by enhancing neurovascular coupling, neuronal differentiation, neurite outgrowth, and mitigating neural injuries [56, 57•, 58, 59].

Excessive circulating Ang II following ARB use can be cleaved into Ang IV by aminopeptidases A and N (AP-A/AP-N) and bind to AT4R to take action. Similar to Ang II/AT2R, Ang IV/AT4R activation is neuroprotective by improving cerebral blood flow, reducing inflammation and oxidative stress, and increasing the release of dopamine and acetylcholine. Its activation is also associated with enhanced long-term potentiation, improvement of memory consolidation and retrieval, and learning ability [59, 60••, 61].

ARBs also reputedly increase the activity of ACE2, a vasodilator peptide that downregulates Ang II levels by degrading it to a heptapeptide, Ang-(1–7). Preclinical evidence suggests that Ang-(1–7) binding to the Mas receptor exerts anti-inflammatory, anti-ischaemic, vasodilatory, and neuroprotective effects [59]. In particular, activation of Ang-(1–7)/MasR can reduce the deposition of β-amyloid and phosphorylated tau and facilitate the release of intercellular messengers, such as prostanoids and nitric oxide, which are important factors in maintaining learning ability and memory [62, 63]. In humans, plasma Ang-(1–7) level is reduced and associated with cognitive function in AD patients [64]. ACEi has no noticeable effect on the expression of ACE2 and Ang-(1–7) in humans, as seen in previous studies [65–68]. In addition, a handful of preclinical studies revealed that ACE2 per se can convert β-amyloid 43, an early-depositing β-amyloid species that contributes to AD pathogenesis, into the less neurotoxic β-amyloid 40 with neuroprotective effects [69].

The “Angiotensin hypothesis” has also garnered support from many clinical studies. Thiazides and dihydropyridine CCBs were demonstrated to increase Ang II-mediated activity by increasing renin and have been shown to be neuroprotective [70, 71]. In contrast, β-blockers and most non-dihydropyridine CCBs can downregulate Ang II expression by reducing renin production and could be potentially harmful. In a post hoc analysis of the PreDIVA Trial, Schroevers et al. [71] assessed the association between the use of Ang II-stimulating anti-hypertensive medications (ARBs, dihydropyridine CCBs, and thiazides) versus any other anti-hypertensive classes and the risk of incident dementia among 1907 community-dwelling adults over a middle- and long-term follow-up. They found that the use of Ang II stimulators was associated with a 32% decreased risk of dementia over a 7-year follow-up, which attenuated to 20% with over a decade of extended follow-up. Notably, the use of ARB and dihydropyridine CCBs was associated with a 46% and 48% reduced risk of dementia over 7 years, respectively, but was attenuated to 25% and 27% over a decade. A post hoc analysis of the SPRINT-MIND trial yielded similar results, with Ang II stimulators associated with a 25% lower risk of dementia compared with other anti-hypertensive medications [72••]. A network meta-analysis of 7 randomized trials and 15 observational studies found that ARBs and CCBs were associated with significantly reduced risk of dementia compared to ACEis and β-blockers [15]. More recently, in a cohort of older adults (*n* = 5047) residing in residential care, Marcum et al. found a significantly lower incidence of cognitive impairment among users of Ang II-stimulators compared to users of Ang II-inhibitors over a median 5.4-month follow-up [73]. Table 2 summarizes recent studies comparing Ang II-stimulating with Ang II-inhibiting anti-hypertensive medications.

**Table 2:** Studies comparing Ang-II stimulators with Ang-II inhibitors

Authors	Study type	Study population	Mean (SD) age	No. of participants	Follow-up duration	Intervention/comparator	Outcomes	Results
Schroevers et al. (PreDIVA trial) [71]	Post hoc analysis of RCT	Community-dwelling older adults without dementia	74.5 (2.5)	1909	6.7 years	Intervention: Ang II stimulator Control: Ang II inhibitor	Dementia	Adjusted HR 0.55; 95% CI, 0.34–0.89
Marcum et al. (SPRINT trial) [72••]	Post hoc analysis of RCT	Adults 50 years or older with hypertension and without diabetes, stroke, or dementia	67.7 (11.2)	8685	4.8 years	Intervention: Ang II stimulator Control: Ang II inhibitor	Amnestic MCI or probable dementia; amnestic MCI; probable dementia	Amnestic MCI or probable dementia: adjusted HR: 0.76; 95% CI, 0.66–0.87 Amnestic MCI: adjusted HR, 0.74; 95% CI, 0.64–0.87 Probable dementia: adjusted HR, 0.80; 95% CI, 0.57–1.14
Marcum et al. [73]	Retrospective cohort study	Long-term care residents aged ≥ 65 years admitted to a Veterans Affairs nursing home	74 (8)	5047	5.4 months	Intervention: AT2R/AT4R stimulator Control: AT2R/AT4R inhibitor	Cognitive impairment	Adjusted HR, 0.83; 95% CI, 0.74–0.93), *p* < 0.01
Marcum et al. [74••]	Respective cohort (US Medicare Insurance)	Adults aged 65 years or older with incident hypertension	73.8 (6.3)	57,773	6.9 years	Intervention: AT2R/AT4R stimulator Control: AT2R/AT4R inhibitor	Alzheimer’s disease and related dementia (ADRD); VaD	ADRD: adjusted HR, 0.84; 95% CI, 0.79–0.90 VaD: adjusted HR, 0.82; 95% CI, 0.69–0.96

Individuals using ARBs, DiCCBs, or thiazide(-like) diuretics were categorized as angiotensin II-stimulating anti-hypertensive users. Individuals using non-DiCCBs, β-blockers, and ACEis were categorized as angiotensin II–inhibiting anti-hypertensive users

*ACEi* angiotensin-converting enzyme inhibitor, *ARB* angiotensin-receptor blocker, *AT2R* angiotensin 2 (Ang II) receptor, *AT4R* angiotensin 4 (Ang IV) receptor, *CI* confidence interval, *DiCCB* dihydropyridine calcium channel blocker, *HR* hazard ratio, *MCI* mild cognitive impairment, *RCT* randomized controlled trial, *VaD* vascular dementia

### Other Possible Mechanisms

Several ARBs, including telmisartan, irbesartan, and candesartan, have been found to modulate the activity of the peroxisome proliferator-activated receptor (PPAR-γ). PPAR-γ is a neuroprotective receptor that can regulate metabolism and reduce inflammation and insulin resistance [54••, 75, 76]. Activation of PPAR-γ reduces inflammation and oxidative stress, improves brain function and metabolism, alters amyloid precursor protein (APP) processing, and reduces β-amyloid accumulation, consequently ameliorating brain cell injury and cognitive function decline. ARBs can also indirectly elevate PPAR-γ expression by blocking AT1R, which is inversely linked to PPAR-γ expression level [77]. Among all ARBs, telmisartan has the strongest agonistic effect on PPAR-γ. By leveraging the National Health Insurance Research Database, a Taiwanese study [78] found that the dementia risk was lower in telmisartan users compared with other ARB users (hazard ratio, 0.72; 95% CI, 0.53 to 0.97; *p* = 0.030). The authors attributed this to the greater effect of telmisartan on increasing PPAR-γ expression.

ARBs may also directly reduce the expression of pro-inflammatory factors, including LPS, IL-1β, and transforming growth factor β (TGF-β), followed by the activation of pro-inflammatory signals, further ameliorating brain cell injuries [79–81]. In contrast, ACEis elevate the level of bradykinin, a pro-inflammatory plasma peptide associated with an increased risk of cognitive impairment, whereas ARBs do not [82]. Studies of animal models found that bradykinin infusion in the brain of mice can lead to significant learning and memory deficits through oxidative stress and synaptic dysfunction, as well as inducing AD-like tau hyperphosphorylation [83]. Conversely, blockade of bradykinin with an antagonist or genetic deletion of the bradykinin B1 receptor can improve cognitive deficits by reducing neuroinflammation and deposition of β-amyloid in AD mice [84, 85]. Elevated bradykinin levels were also seen in the cerebrospinal fluid of mice after cerebral injection of β-amyloid [86].

**Fig. 1:**
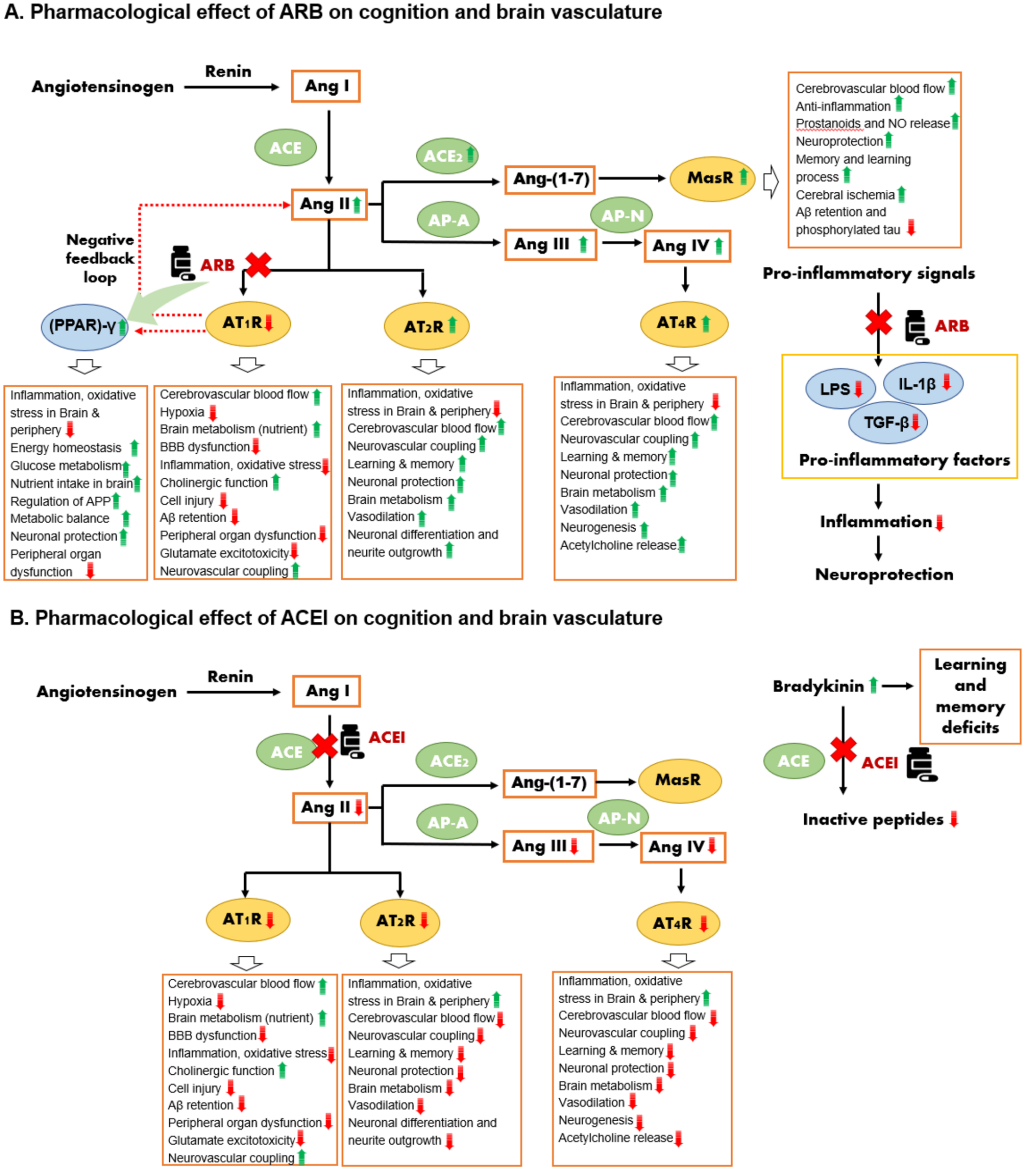
Putative mechanisms by which the ARB and ACEis affect neurodegeneration. Renin cleaves angiotensinogen to form angiotensin (Ang) I, which is subsequently converted to Ang II by the angiotensin-converting enzyme (ACE). Ang II exerts physiologic effects by binding to AT1 or AT2 receptors (AT1R or AT2R). Ang II can be further cleaved to Ang IV by AP-A and AP-N, which bind to AT4R to take effect. ARBs selectively prevent Ang II from binding to AT1R without affecting ACE activity. Blocking AT1R activation can in turn increase Ang II expression upstream as a result of negative feedback regulation, and this will result in an upregulation of Ang II/AT2R and Ang IV/AT4R signalling to exert pleiotropic neuroprotective benefits such as improvement of episodic memory. Additionally, ARBs may increase the activity of ACE2, which can convert Ang II to Ang-(1–7). Ang-(1–7) can bind to the Mas receptor to exert neuroprotective benefits. Several ARBs, including telmisartan, irbesartan, and candesartan, can also modulate the activity of PPAR-γ to exert benefits. In addition, ARBs were found to ameliorate inflammation by downregulating the expression of pro-inflammatory factors such as LPS, IL-1β, and TGF-β to pro-inflammatory signals and provide neuroprotection. ACE inhibitors (ACEis) directly inhibit ACE activity, thereby inhibiting Ang II production and its derivatives, AT1R, AT2R, and AT4R. Inhibiting AT1R activity is beneficial for cognition, while inhibiting AT2R and AT4R are harmful for cognition. ACEis have no obvious impact on ACE2 expression and thus do not impact the formation of Ang-(1–7). ACEi can however increase the levels of bradykinin by preventing it from degrading to inactive peptides and this will contribute to learning and memory deficits. Abbreviations: ACE, angiotensin-converting enzyme; ACEi, angiotensin-converting enzyme inhibitor; APA, aminopeptidase A; APN, aminopeptidase N; APP, β-amyloid (Aβ) precursor protein; ARB, angiotensin receptor blocker; AT1R, angiotensin II type-1 receptor; AT2R, angiotensin II type-2 receptors; AT4R, angiotensin II type-4 receptors; BBB, blood–brain barrier; IL-1β, interleukin 1β; LPS, lipopolysaccharides; PPAR-γ, peroxisome proliferator-activated receptor; TGF-β, tumor growth factor-β

Figure 1A illustrates the major putative mechanisms underlying the proposed association between ARBs and neurocognitive protection. Figure 1B summarizes the putative mechanisms for the proposed effects of ACEi use on neurocognition. In brief, ARBs exert protective effects by directly blocking the Ang II/AT1R pathway and stimulating neuroprotective Ang II/AT2R, Ang IV/AT4R, and Ang-(1–7)/MasR pathways, while ACEis block the Ang II/AT2R and Ang IV/AT4R pathways that counterbalance the neurocognitive benefits of blockage of the Ang II/AT1R pathway. ARB use has also been linked to the activation of PPAR-γ, a biomarker associated with enhanced brain function and metabolism, minimized expression of pro-inflammatory factors, and reduced β-amyloid accumulation.

## Potential Modifiers of the Putative Beneficial Effects of ARBs

The mechanisms underlying the role of ARBs in dementia and cognition decline remain inadequately understood. Mechanistic studies are needed to provide deeper insights to support the proposed mechanisms, and more clinical studies are needed to verify the postulated benefits of ARBs in humans. Identifying potential modifiers of these putative benefits may help elucidate the inconsistent results of previous studies. In the following section, we summarize the findings from existing studies conducted in diverse populations and identify possible modifiers of ARB actions.

### Blood-Brain Barrier (BBB) permeability

The neuroprotective effects of specific anti-hypertensive classes or agents may be mediated by their ability to penetrate the BBB. Some, but not all, epidemiologic studies found a stronger inverse association between BBB-crossing RAS anti-hypertensive medications and dementia risk among individuals with and without cognitive impairment [87]. In mouse models, hippocampal neurodegeneration induced by β-amyloid accumulation was entirely rescued, and neurons were protected by brain-penetrant ACEi (captopril) and ARB (losartan) [88]. A cohort study [89•] conducted on patients with AD reported the slowest decline in delayed recall performance among users of BBB-crossing ARBs, followed by non-BBB-crossing ARBs and BBB-crossing ACEis when compared to non-BBB-crossing ACEis. The Alzheimer’s Disease Neuroimaging Initiative (ADNI) study of individuals without dementia found that BBB-crossing ARBs were associated with better memory performance and a lower volume of white matter hyperintensities when compared to other anti-hypertensive agents [90]. One study indicated that BBB penetrability only modifies the potentially neuroprotective effect of ARBs, not ACEis, a finding that warrants further investigation [50].

### Cognitive Impairment and Genetic Risk Factors

The neurological effects of ARBs may differ in populations with different initial dementia risks. Most previous studies were conducted on individuals initially free of any neurocognitive or cerebrovascular disorders. A network meta-analysis of 19 randomized trials in a total of 18,515 individuals without prior cerebrovascular events found that ARB use was associated with decreased risks of overall cognitive decline and dementia compared with other anti-hypertensive classes while maintaining similar effectiveness in BP control [10].

The question of whether ARBs also provide benefits to individuals with mild cognitive impairment (MCI) in terms of preventing progression to dementia as well as to those diagnosed with AD in terms of improving prognostic outcomes remains inconclusive. Some studies showed positive findings in this regard. Ramipril had no effect on β-amyloid levels in CSF [91], while ARB use compared with no use or other anti-hypertensive class use was associated with significantly reduced tau and phosphorylated tau expression and ameliorated amyloid pathology among MCI patients [92•]. Deng et al. demonstrated that ARB use in MCI individuals was associated with a decreased risk of progression to dementia compared with ACEi use, other class use, and no anti-hypertensive medication use [93••]. Similar results were seen in an AD cohort (*n* = 1689), where ARB users exhibited better preservation of memory, attention, and psychomotor processing speed than ACEi users [89•]. Subsequent subgroup analyses suggested that the ARB-associated benefits were limited to APOE ε4 non-carriers. Another recent cohort study of a mixed older population, including those with normal cognition, MCI, and dementia [28•], found that in individuals with normal cognition, ARBs were only beneficial in the absence of APOE ε4. Among individuals with AD/MCI and accumulation of β-amyloid, there was no difference between ARBs and ACEis in rates of overall mid/late-stage β-amyloid accumulation, regardless of APOE ε4 carrier status.

### CVD as a Risk Factor

CVD is an important risk factor and contributor to the progression of dementia. In addition to its BP-controlling properties, ARBs are commonly utilized for the treatment of various cardiovascular conditions, such as heart failure. ARBs have potential neuroprotective effects irrespective of patients’ history of CVD. The ONTARGET trial studied 25,620 older patients (mean age of 66 years) with coronary heart disease and mildly elevated systolic BP (142 mm Hg) [34••]. The incidence of combined neurological outcome, stroke, and cognitive impairment was 10% lower in the telmisartan group compared to the ramipril group, despite a mere 0.9 mmHg lower systolic BP with telmisartan. In contrast, when examining the same outcome, the TRANSCEND trial (*n* = 5926; mean age: 67 years), which compared telmisartan to placebo in individuals intolerant to ACE inhibitors, did not report a significant association of cognitive function with telmisartan [34••]. Furthermore, in an older cohort initially free of cognitive problems but with a history of ischemic heart disease, BBB-crossing ARBs were associated with a lower risk of AD when compared with non-users. This relationship appeared to be dependent on dosage and treatment length [50]. These data collectively suggest that ARBs may provide superior protection against AD compared to other anti-hypertensive classes in CVD patients.

While the effects of ARBs and other classes on vascular dementia in individuals with CVD may be similar, as they demonstrate comparable efficacy in BP reduction, the magnitude of the reduction in AD risk with ARBs may surpass the reduction in vascular dementia risk. A decline in visuospatial skills, executive function, and attention is more characteristic of vascular dementia, while a decline in episodic memory and language ability is more pronounced in AD patients [94]. Previous studies have consistently reported the benefits of ARBs on memory and language, suggesting their direct impact on AD pathology [90, 94, 95•]. Patient populations with high CVD risk in whom the ratio of vascular to AD pathology is increased may be less likely to obtain more benefit from ARBs compared with other classes.

### Duration of ARB Use

The duration of ARB use is a crucial aspect that must be considered in dementia research. Dementia can develop insidiously over many years and take decades to manifest clinically, such that if ARBs confer any protection, the benefits may become apparent only after long-term use. Short-term studies on anti-hypertensive medications have predominantly focused on assessing cognition, while longer-term studies have explored dementia outcomes. A small-scale trial of adults aged over 55 years with mild cognitive impairment and hypertension found that 1-year treatment with candesartan was associated with a slower decline in executive function and episodic memory compared with lisinopril, despite similar BP control between the two treatment arms [48]. A meta-analysis has revealed the anti-hypertensive medication-associated benefits of dementia may increase with longer follow-up [15]. A prospective cohort study with a decade of follow-up found that anti-hypertensive medication use was associated with a 5% reduced risk of incident dementia per year, with the strongest association observed for ARBs, showing a 15% risk reduction per year [96]. A population-based study in Taiwan (*n* = 49,062) matching users of ARBs with non-ARB anti-hypertensive medications found that, compared with other anti-hypertensive medications, the HRs of incident dementia with ARB use < 4 years and > 4 years in duration were 0.62 (95% CI 0.57–0.66) and 0.34 (95% CI 0.30–0.39), respectively [42]. An early clinical trial involving 4954 subjects aged 70–89 years old found no significant difference in the change of cognitive function measured by the mini-mental state examination over time and dementia risk between candesartan and placebo [97]. The authors attributed these non-significant findings to the short-term follow-up period (mean 3.7 years) and the modest reduction in BP achieved with candesartan (systolic BP: − 3.2 mm Hg, diastolic BP: − 1.6 mm Hg).

### Dosage

The magnitude of ARB-associated neuroprotection has been shown to be dose-dependent. Li et al. followed 819,491 older American veterans with CVD for 4 years and reported a significant association between the use of ARB and lower incidence and progression of AD and dementia when compared with ACEi and other cardiovascular drugs, and the strength of the association increased with a higher ARB dose [39••]. Another large-scale case–control study reported dose-dependent inverse associations between ARB and AD [32]. Thus, a careful balance of countering the risk of using high-dose ARBs with its benefits is important in clinical practice.

## Clinical Implications and Remaining Questions

Existing evidence regarding the overall effects of anti-hypertensive medication use on cognitive decline and dementia, as well as determining which specific class or agent provides the greatest neuroprotection, remains inconclusive. A significant body of evidence supports the potential beneficial effects of ARBs compared with other classes. However, most studies supporting ARBs’ ability to lower dementia risk have relied on preclinical evidence and observational data. To inform evidence-based clinical practice and relevant treatment guidelines, it is imperative to have high-quality evidence derived from large-scale, meticulously conducted prospective randomized controlled trials with long-term follow-up. An important clinical question arises regarding whether ARBs should replace ACEis in populations at high risk of neurocognitive disorders, given that both medications have similar indications and clinical uses, often leading to their interchangeable prescribing. While awaiting more robust evidence and consideration of dementia as an indication for ARB use in hypertensive individuals within clinical guidelines, it may be advisable to recommend ARBs over ACEis for those with indications for RAS inhibitors who express concern about or susceptibility to cognitive decline and dementia. Additionally, compared to ACEis, ARBs confer additional safety benefits, including a lower risk of cough, angioedema, pancreatitis, and gastrointestinal bleeding [98].

Future research concerning the neurocognitive effects of ARBs and other anti-hypertensive classes should incorporate additional elements such as blood biomarkers, brain imaging scans, and genetic factors like APOE ε4 carrier status and familial aggregation. These measures can contribute to a deeper understanding of the underlying mechanisms driving the observed associations. Consideration should also be given to the pre-existing risk of cognitive decline and dementia in individuals, the duration and intensity of treatment, and the pharmacological properties of specific ARB agents (e.g., BBB permeability). These factors are important as they can potentially modify the associations of interest. Studies relying solely on baseline exposure data may be susceptible to biases, particularly long-term follow-up studies where the data are less representative of actual medication use. This issue is especially pertinent among older individuals, as their BP can vary, and changes in anti-hypertensive medication usage are common. To ensure the reliability and robustness of the results, careful consideration and elimination of potential biases commonly encountered in epidemiological studies, such as reverse causality, indication bias, biases arising from unmeasured or unobserved factors, time-varying treatment exposure, and uncertainty regarding dosages, are necessary.

## Conclusion

ARBs may outperform other anti-hypertensive classes in preventing or delaying cognitive decline and dementia and, therefore, offer a promising therapeutic avenue. However, the inconsistent findings observed in previous studies regarding the neuroprotective effects of ARBs emphasize the need for future long-term follow-up studies involving larger and more diverse populations to yield more robust evidence in this context. Gaining a better understanding of the intricate mechanisms involved in the potential neurocognitive benefits of ARBs, mediated through interconnected RAS pathways and other mechanisms, can offer valuable insights into identifying molecular targets aimed at preventing and treating dementia and cognitive decline.

## Data Availability

No data were used for drafting this manuscript.
